# The immunomodulatory properties of low-level ionizing radiation as a potential treatment for COVID-19’s life-threatening symptoms

**DOI:** 10.1186/s40001-023-00999-7

**Published:** 2023-02-11

**Authors:** Soha M. Hussien

**Affiliations:** Radiation Safety Department, Nuclear and Radiological Safety Research Center, Egyptian Atomic Energy Authority, Cairo, Egypt

**Keywords:** COVID-19, Immunomodulatory, Anti-inflammatory, Radiotherapy, Low-dose

## Abstract

Public health experts are looking into the current coronavirus outbreak to see if there are any ways to prevent potentially fatal symptoms. Low-Dose Radiotherapy (LD-RT) induces anti-inflammatory cytokine responses that act as a counterweight to pro-inflammatory cytokines, potentially providing therapeutic benefits for COVID-19-related diseases associated with significant morbidity and mortality. This study will look into positive immuno-radiological reactions to see if they are feasible, practicable, and effective in lowering the critical inflammatory condition of the crucial stage COVID-19. This study aims to investigate the use of low-dose lung radiation in bacterial and viral pneumonia, as well as to provide a treatment plan for COVID-19-associated pneumonia. This article discusses the evidence for and against LD-RT theories in COVID-19 patients. The use of LD-RT at various stages of COVID-19 appears to be beneficial, with fewer side effects than other currently being studied treatments.

## Introduction

The current outbreak was caused by SARS-CoV 2, also known as COVID-19, entering China in December 2019. Three CoV epidemics have afflicted humanity in the last two decades [1, 2]. The study is still in its early stages, and many published papers may have received insufficient peer review. However, it must be matched by a willingness to share new information to understand better.

The SARS-CoV-2 virus spread quickly worldwide, leaving many countries unprepared for a pandemic threat [3]. Infected patients may develop asymptomatic, moderate to severe upper respiratory illness, pneumonia, ARDS, or die [4]. Most of the ICU patients will require respiratory support [5]. Pneumonia and ARDS treatment are critical during high-mortality periods, but the new pneumonia therapy strategy had to be rethought. Previous research indicates efficacy at doses less than 1 Gy, close to the dose criteria for acute and chronic inflammatory and benign degenerative disorders [4]. In the early twentieth century, X-rays were commonly used to treat pneumonia. Prior research found that LD X-ray reduced pneumonia mortality by 30–10% on average. According to previous research, the development of an LD-RT-induced anti-inflammatory phenotype may account for the observed effects [6].

Irradiation less than 1 Gy into the lungs of a COVID-19 patient reduces inflammation and life-threatening symptoms [7], with a more significant reduction in inflammatory cytokines [8], LD has been shown to have anti-inflammatory properties. Despite the pro-inflammatory effects of high doses [9, 10]. This study investigates the range of data supporting LD-RT use and inherent conflicts in using LD-RT in COVID-19 patients.

## The use of LD radiation in the treatment of bacterial and VP in the chest RT before penicillin

### A. Studies that support the theory

Animal pneumonia research has revealed a similar response, with X-ray treatment significantly lowering mortality from around 30% to 5–10% in rats, guinea pigs, cats, and dogs. Individual attempts were made in the early twentieth century to use IR to cure various non-cancer ailments. Calabrese and Dhawan emphasize that LD-RT can improve current thinking by reviewing the findings of 19 studies on the efficacy of LD X-ray RT for pneumonia. Eight hundred sixty-three people were studied, with 717 demonstrating a significant clinical response after three days of treatment [6].

Case reports constituted the majority of historical human research, with six containing comparison control groups. The death rate in the scientific community was around 30%, which was comparable to the national average. Another study found that nearly 4000 people experienced significant relief from their pulmonary symptoms [3]. A review of 15 studies examined 863 patients with varying etiologies, including two cases of viral origin, using LD X-rays. IR has been shown to have good clinical responses, with side effects appearing within 1–3.5 days of treatment and decreasing mortality [9].

### B. Studies did not support the theory

Oppenheimer saw 56 patients in 1943 who received 0.35–0.9 Gy of LD thoracic RT for VP. The available data are limited to a few cases of VP being treated in this manner. These investigations are hampered by conflicting variables [11]. Dr. Oppenheimer discovered that thoracic RT could treat VP in the early stages of respiratory disease. The case series is fascinating because those who received treatments improved quickly, whereas those with symptoms for a week were resistant. Two patients reported that their symptoms worsened after RT. A 1943 study of 155 VP patients found 12 fever, sore throat, and chills but no dyspnea [12]. The recovery time is estimated to be 12 days. There was no statistically significant difference between RT patients and non-RT patients. Kirsch et al. fault the authors for failing to provide sufficient information on how 15% of patients received RT. Because of the selection bias, individuals in the RT-treated experiment may have been sicker for a shorter period [5].

According to the findings of these studies, RT should be administered early in the inflammatory process. There is no discernible effect of the dose on the effectiveness of 0.1–1 Gy. A cure rate of more than 90% is achieved when treatment is initiated within 24 h of the onset of inflammation. However, if given at the peak of inflammation, the cure rate drops to 50% [9, 13].

**Fig. 1:**
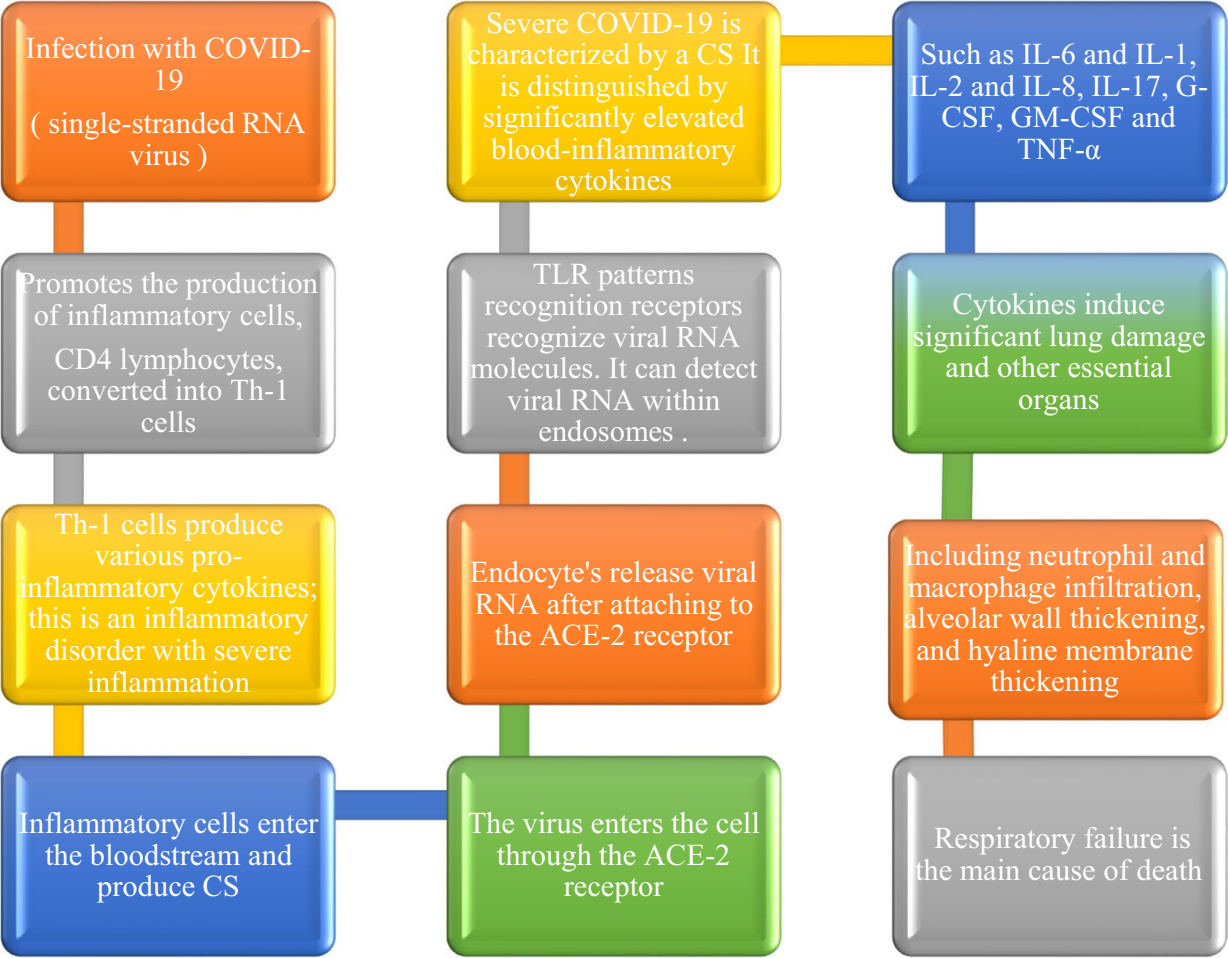
The pathogenesis of SARS-CoV-2 and its immunological consequences

Salomaa et al. used three radiobiological assays in their research. Fried [14] used guinea pigs, Lieberman et al. [15] used dogs, and Dubin et al. [16] used mice, the first two for bacterial pneumonia and the second for VP. The majority of recovered animals in the third population had been irradiated three days after bacterial infection, compared to an average survival of 2.1 days in control mice. (Infected but not irradiated): this supports the experimental selection bias [17] (Fig. 1).

*Many COVID-19 cytokines have been linked to respiratory illnesses. Inflammatory cells enter the bloodstream and produce CS, which damages lung epithelial and alveolar cells, among other organs, rapidly “*[18–20].*” Type I interferons are produced in COVID-19 infections, followed by a pro-inflammatory cytokine cascade. In minor cases, the immune system helps manage infections and promotes healing. In patients with severe infections, lymphopenia (lower CD-4, CD-8, B, and NK cells) is typical* [21]*. The possibility of CS as a cause of severe influenza-associated viral syndromes has been proposed. This occurrence has been linked to many other pharmaceutical medications, the vast majority of which are immunomodulatory. CS has been proposed as a possible cause due to the significant T-cell activation* [22]*.*

## COVID-19 therapy necessitates LD-RT research

The novel CoV has spread rapidly, causing severe disease in many people and putting major healthcare systems under strain. According to some studies, COVID-19 patients should receive LD (less than 1 Gy) thoracic RT to improve their chances of survival [7]. Other studies, however, say the evidence for LD-efficacy RTs is insufficient to justify the higher risk of human pneumonia being treated [5]. There are no effective treatments for COVID-19. A new study is needed to determine whether LD-RT can be used to treat life-threatening symptoms.

### A. How widely spread is the LD-RT?

Kirkby et al. estimate that exposure of 0.3–1 Gy is possible with a megavoltage system. When using LD, conventional RT toxicity is reduced [7]. Most medical facilities, including emergency departments, outpatient clinics, and hospitals, use X-ray technology. Using X-ray equipment is less expensive than taking prescription medications, especially in developing countries with limited infrastructure. Patients on ventilators can be treated in isolation rooms or ICUs [3].

## The mode of action of the LD-RT

Calabrese et al. report that LD-RT induces a highly integrated, sophisticated, and systemic response to the M-2 anti-inflammatory phenotype, including macrophage polarization. In other studies, the anti-inflammatory phenotype M-2 has been found to inhibit leukocyte and polymorphonuclear cell adhesion, reactive oxygen species, nitric oxide, TNF-α, and endothelial cell adhesion. Following the M-2 phenotype, LD-RT is accompanied by increased heme oxygenase, anti-inflammatory cytokines IL-10, and transcription factor activation [8, 23, 24], increased TGF-β1 synthesis, and developed T-regulatory cells [24–26]; as a result, the LD-RT pathway is validated both in vivo and in vitro by the onset of anti-inflammatory properties [27].

## LD-RT’s role in the treatment of related conditions

RT is a standard treatment for non-malignant illnesses in many countries. Every year, 5000 people in Germany receive non-cancerous irradiation at more than 300 RT centers. Non-malignant conditions account for 10–30% of all RT patients, which is unusually high compared to most other countries. There is a scarcity of information on RT for pneumonia, and more research is required into this condition’s treatment [28]. The findings suggest that inflammation plays a significant role in the severity of COVID-19 and that IL-6, TNF-α, and IL-8 may be promising treatment targets. In hospitalized COVID patients, IL-6 is a strong predictor of respiratory failure and CS, resulting in a patient's death [29]. IL-6 plays an essential role in treating diseases, such as collagen-induced arthritis, experimental encephalomyelitis, and systemic lupus erythematosus. According to one study, protein plays a vital role in the body's immune system [30]. According to a new study, IL-6 levels in rat models of human disease were elevated in most cases and significantly reduced by LD-RT [24, 26].

With over 37,000 patients treated each year, Germany has been the most enthusiastic promoter of RT for benign disorders [31, 32]. In 2002, the German working group on benign disease RT issued a consensus statement on prospective indications and pharmacological therapy recommendations. It was agreed that LDs should be treated with acute and chronic inflammatory illnesses and painful acute and joint degenerative diseases [33]. In these inflammatory diseases, the LD-RT process is finely regulated by leukocyte–endothelial cell interactions and the activity of inflammatory mediators and adhesion molecules released by various peripheral blood cells, such as leukocytes, neutrophils, and macrophages [9].

Ab El-Fatah et al. looked into the effects of LD-RT on the inflammatory environment of joint, kidney, liver, and hematological diseases. They discovered that LD- RT could cause hormesis-like reactions. After treatment, total leukocyte counts, serum creatinine, and serum liver enzymes decreased significantly (p < 0.01). Treatment was suggested for patients with multi-system pro-inflammatory illnesses, such as chronic renal disease [34].

Calabrese et al. investigated the use of infrared radiation in the treatment of inflammatory diseases. They compiled information from over 37,000 patients suffering from 13 different diseases. RT resolved all 13 instances with a 90 percent response rate. Over a dose range of 0.3–1.5 Gy, this exceptional and consistent RT efficiency was achieved [35]. LD-RT has been shown to have anti-inflammatory properties for nearly a decade [9, 36, 37]. LD-RT is widely used in Germany, although it is still infrequently used in other countries. Because of the possibility of delayed toxicity at much larger RT doses attributable to LD-RT, LD-Treat may only be used as a last resort in benign circumstances [31, 32]. The limited use of RT in benign conditions is due to the risk of carcinogenic IR and a lack of controlled trials investigating this application. Obsolete RT methodologies and data from Hiroshima and Nagasaki, where radiation exposure has spread widely throughout the body, provide evidence of cancer risk. According to the study, the risk of RT cancer for a mild condition is low when using the current recommended procedures [38]. The COVID-19 study's suggested dose is lower than what is typically used for minor diseases.

## Recommendations for LD-RT dose in COVID-19 patients with ARDS

Genard et al. 2017 investigated macrophage polarization's molecular mechanisms using multiple mouse models and human cell lines. The findings revealed a three-phase response curve in which low- and high-dose RT polarized M2 (anti-inflammatory), but moderate-dose RT (1–10 Gy) polarized M1 (pro-inflammatory phenotype) [39]. The M1 and M2 phenotypes are not absolute in single cells or cell populations but instead show a combination. This theory holds that pro- and anti-inflammatory phenotypes can coexist and that the radiation dose determines the final phenotype [35]. Klug and colleagues discovered that the tissue microenvironment influences cell LD-RT polarization [40]. Roedel and colleagues state that linear energy transfer radiation has shown promise as a treatment option for COVID-19 patients with VP. Because of attenuation through the chest wall, the mean dose ranges from tens to one Gy.

Scientists have been studying the fundamental principles underlying the efficacy of these doses for the past three decades. Indeed, in vitro and in vivo studies have revealed a complicated relationship between LD-RT and inflammatory pathways. Numerous studies have been carried out to investigate methods for modulating the inflammatory properties of leukocytes, macrophages, fibroblasts, and endothelial cells and their cytokine/chemokine production and growth factors [10, 41]. So far, the investigated pathways have similar dose–response relationships, with substantial effects ranging from 0.3 to 0.7 Gy, previously recognized as the most potent in clinical situations, such as pneumonia treatment. Despite the scarcity of experimental or preclinical data on LD-RT testing in COVID-19 respiratory distress patients, a single 0.5 Gy treatment, similar to the previous proof, may be recommended.

For the best therapeutic efficacy, Calabrese et al. recommended a dose range of 0.2 to 2 Gy. According to the researchers, this tailored LD-RT appears most effective during acute illness when cytokines are at their peak. COVID-19 patients with CS should receive a single total dose of 0.3–0.5 Gy, according to the authors. Furthermore, it significantly reduces the possibility of any negative long-term consequences [35].

It was recently demonstrated that LD-RT could treat COVID-19 pneumonia with a single acute dose of 0.3–1 Gy of low radiation to the lungs while causing no natural tissue damage. Ghadimi-Moghadam et al. [42] propose that COVID-19 pneumonia be treated with a few mGy priming doses followed by a single 0.25 Gy dose. In Ramsar, Iran, the maximum natural background radiation exposure is 0.26 Gy/year. Clinical LD-RT investigations are recommended for COVID-19 pneumonia by both Ghadimi-Moghadam et al. and Kirkby and Mackenzie [7, 42].

### A. The radiation time in COVID-19 patients

Although LD-RT does not affect virus pathogenicity, it does improve antiviral immune response efficiency [43]. Lung LD-RT treatment significantly reduces inflammation and disease severity in early to mid-stage SARS-Cov2 infection. LD-RT has also been shown to boost virus-specific immune features, like NK cell activity and interferon production [44]. According to a previous study, LD-RT may be less effective in the chronic stage of the disease, as defined by CS, than in the early stages [45]. It emphasizes the importance of precisely controlling the irradiation time.

### B. The side effects of LD-RT

Radiation causes lung cancer in both men and women, according to research. According to Kirkby and Mackenzie's research, females and smokers are more likely than non-smokers and males to develop lung cancer. Acute lung doses of 0.3–1 Gy are recommended [7]. There is no need to deal with acute, early typical tissue damage at 0.5 Gy. According to the International Commission on Radiological Protection, such risk assessments should not be used in medical exposure scenarios or individual patients. The risk of developing cancer is low at 0.5 Gy[5]. Dhawan et al. do not provide any computations, implying that the risk is low. There is no pharmaceutical treatment that can provide accurate estimates of lifetime risk. Even after adjusting for the risk estimates of Kirsch et al., the overall lifetime risk of this life-saving treatment is 1–2% [3, 46]. Researchers warn that COVID-19 patients must be chosen with caution. According to the researchers, some of the experimental medications used may have serious side effects. They add that the study emphasizes the importance of careful patient selection [47].

### C. Safety

Radiation, according to experts, has a more local effect on the body than other pharmacological treatments with a sizeable systemic effect. An LD-RT is required for effective treatment. According to conservative research estimates, the risks for lung cancer patients are less than 1%. The risk of dying from lung cancer is less than 1% [48]. At doses of up to 0.5 G, the drug, LD-RT, can cause mild pressure [49, 50]. Numerous experimental treatments are being tested, including clinical trials for whole-lung LD-RT, defined as 0.3 to 1.5 Gy delivered in a single fraction and is less than the occupational exposure limit of 50 mSv/year and the general public exposure limit of 1 mSv/year [51, 52].

## Summary and conclusion

Researchers believe that more research into the efficacy of complete LD-RT would benefit from treating inflammatory/infectious illnesses, such as COVID-19 pneumonia patients based on its proposed mode of action to reduce the extensive inflammatory repercussions of CS and potentially reduce death. LD-RT has anti-inflammatory effects on systemic inflammation regardless of where the inflammation is located. Clinical and analytical evidence on VP, dose levels, and irradiation timing is lacking. More research is needed to determine whether RT benefits COVID-19 patients suffering from hypoxia.

## Data Availability

Available.

## Abbreviations

SARS-CoV 2: Severe acute respiratory syndrome coronavirus 2
COVID-19: Coronavirus disease 2019
CoV: Coronavirus
ARDS: Acute respiratory distress syndrome
ICU: Insensitive care unit
Gy: Gray
LD: Low-dose
RT: Radiotherapy
VP: Viral pneumonia
IR: Ionizing radiation
CD: Cluster of differentiation
TH: T Helper cell
CS: Cytokines storm
ACE-2: Angiotensin-converting enzyme 2
TLR: Toll-like receptors
IL: Interleukin
G-CSF: Granulocyte colony-stimulating factor
GM-CSF: Granulocyte–macrophage colony-stimulating factor
TNF: Tumor necrosis factor
NK: Natural killer
TGF-β: Transforming growth factor beta
mSv: Milliesievert
